# Quantized Residual Preference Based Linkage Clustering for Model Selection and Inlier Segmentation in Geometric Multi-Model Fitting

**DOI:** 10.3390/s20133806

**Published:** 2020-07-07

**Authors:** Qing Zhao, Yun Zhang, Qianqing Qin, Bin Luo

**Affiliations:** The State Key Laboratory of Information Engineering in Surveying, Mapping and Remote Sensing, Wuhan University, Wuhan 430072, China; zhaoqing@whu.edu.cn (Q.Z.); zhangyunmail@whu.edu.cn (Y.Z.); qqqin@lmars.whu.edu.cn (Q.Q.)

**Keywords:** geometric model fitting, quantized residual, linkage clustering, sampling and clustering

## Abstract

In this paper, quantized residual preference is proposed to represent the hypotheses and the points for model selection and inlier segmentation in multi-structure geometric model fitting. First, a quantized residual preference is proposed to represent the hypotheses. Through a weighted similarity measurement and linkage clustering, similar hypotheses are put into one cluster, and hypotheses with good quality are selected from the clusters as the model selection results. After this, the quantized residual preference is also used to present the data points, and through the linkage clustering, the inliers belonging to the same model can be separated from the outliers. To exclude outliers as many as possible, an iterative sampling and clustering process is performed within the clustering process until the clusters are stable. The experiments undertake indicate that the proposed method performs even better on real data than the some state-of-the-art methods.

## 1. Introduction

Traditionally, when dealing with geometric model fitting problems in computer vision, it is considered that there is only one model instance in the data, and the classical method—random sample consensus (RANSAC) [1]—is used to estimate the model. However, in most cases in computer vision, there are actually more than one model instance in the data for most cases. Since real data with multiple instances in computer vision are much more complicated, there is likely to be lots of noise and outliers, and also pseudo-outliers [2] (data belonging to one model are usually outliers to other models). Thus, basing the methods on the single model fitting methods (e.g., sequential RANSAC [3,4] and multi-RANSAC [5]) usually fails when dealing with multiple geometric model fitting problems.

As a matter of fact, the multi-model fitting problem can be considered to be a typical example of a chicken-and-egg problem [6]: both the data-to-model assignments and model parameters are unavailable, but given the solution of one subproblem, the solution of the other can be easily derived. Most of the multi-model fitting methods first generate large amount of hypotheses, and then conduct selection or representation on the hypotheses. Randomized Hough transform (RHT) [7] selects good models for multiple instances by taking the peaks in the histogram built in the parameter space, and residual histogram analysis (RHA) [8] finds the peaks of the residual histogram. The mean shift clustering method introduced in [9,10] works in the parameter space to seek multiple models. Robust preference analysis (RPA) [11] performs symmetric non-negative factorization on the cleaned kernel matrix to extract the most representative models. Meanwhile, J-linkage [12,13] adopts a conceptual representation of points, and through the linkage clustering of the point preferences, it segments the inliers into different models. T-linkage [14,15] uses relaxation of the binary preference function and the soft Tanimoto distance to improve J-linkage for clustering. To find the inlier clusters, one of the crucial problems is how to remove the outliers and noise. J-linkage and T-linkage get rid of outliers and noise by means of an inlier threshold. Kernel fitting (KF) [16] makes use of the sorted residuals of the hypotheses to build the Mercer kernel to elicit potential points belonging to a common structure and, as a result, the outliers and noise can be removed. Adaptive kernel-scale weighted hypotheses (AKSWH) [17] uses a simultaneous fitting and segmentation framework that simultaneously selects good models and segments the inliers, and it can remove the outliers and noise by the iterative Kth ordered scale estimator (IKOSE). Although a framework of simultaneous sampling and multi-model fitting has been proposed [6] to solve multi-model fitting problems, the hypothesis generation and model fitting are processed simultaneously, so this approach is prone to local optima.

Recently, a series of optimization-based methods [18,19,20,21,22,23,24,25] were proposed to solve the multi-model fitting problem, in which [18,19,20,21,22] deal with the multi-model fitting problem as a multi-labelling problem by using energy minimization function and successfully introducing spacial information of the inliers, and [23,24,25] solve the problem by using hypergraphs [26] to describe the relationship of the minimum sampling set and the hypotheses for inlier clustering. However, these optimization-based methods can hardly handle the outliers and need extra inlier threshold or scale estimation techniques.

From the previous relevant work, preference analysis-based methods consisting of conceptual preference [11,12,13,14,15] and permutation preference [16,17,27,28], are the mainstream when dealing with multi-model fitting problems. However, conceptual preference by binarizing the residuals using an inlier threshold in J-linkage [12,13], extremely compresses the differences between models and loses lots of information. Although T-linkage [14,15] uses relaxation of the binary preference function and the soft Tanimoto distance to improve the conceptual preference in J-linkage, increasing the information and keeping the difference between models, which also keeps the differences between inliers belonging to the same model. what’s more, the conceptual preference in J-linkage and T-linkage needs one inlier threshold or one time constant for all the models to eliminate the impact of outliers, which is not appropriate for most cases in multi-model fitting. Permutation preference simply uses of the order number of the sorted residuals as the preference, which is very sensitive to the outliers and noise. Since conceptual preference with binarizing the residuals will lose the differences between models, but the relaxation of the binary preference function will increase the differences between the inliers belonging to the same model. Considering to keep the differences between models and decrease the differences between inliers belonging to one model, the quantized residual preference is proposed in this paper, which make the preference for hypotheses and points by using the quantized value of the residual to select good hypotheses and segment the inliers. In addition, in order to deal with the outliers, we propose dichotomous method by preference linkage clustering of inliers and outliers for each model.

A quantized residual-based two-stage multi-model fitting method is proposed in this paper to take advantage of the similarities between not only the point set, but also the hypotheses. Both stages make use of the quantized residual and contain a linkage cluster process, the difference is that the objects used for clustering are not the same. The first part is model selection, which is designed to cluster similar hypotheses and output several clusters with corresponding inlier sets. The output clusters represent different models in the scene, however the inlier sets only contain a few points which fit the models best. To obtain all the inliers of each model, the second part called inlier segmentation is designed. For the purpose of accurately obtaining all the inlier point sets under the interference of outliers, an alternate sampling and clustering strategy is adopted in the inlier segmentation, which is able to distinguish inliers from outliers.

The model selection part is implemented by a bottom-up merging strategy with quantized residual preference linkage clustering. Firstly, following the classical framework of multi-model fitting, we make use of the spatial information and uniform sampling to generate hypothesis, which we conduct random sampling within subregions of the data space. Next, the hypotheses are weighted by sum of residuals with a fixed number of inliers, and then quantized residual preferences are made for the hypotheses for linkage clustering, which iteratively conducts bottom-up merging of two hypotheses with minimum distances and update the preference with higher weight hypothesis. Finally, good hypotheses with the minimum sum of residuals are then selected as the model selection results.

Unlike most of the current methods, where the inlier segmentation always needs an inlier threshold or a scale estimation technique [17,29,30,31], the inlier segmentation in this paper is conducted by linkage clustering of the quantized residual preference extracted from the corresponding hypotheses residuals. The quantized residual preference for the point representation extracted from the good hypotheses inliers allows robust identification of the inliers, and can separate inliers from outliers quite well. Iterative inlier clustering and hypothesis sampling is performed to make the results more stable and get rid of as many outliers as possible. This process works without an inlier threshold or scale estimator, and can separate the inliers and outliers quite well.

Therefore, the contributions of this paper are three folds: (1) Quantized residual preference is proposed to represent the hypotheses, and the weighted preference similarity is introduced to measure the similarity of two hypotheses, which is used in the adjusted linkage clustering for model selection. (2) Quantized residual preference is proposed to represent the points in linkage clustering to segment inliers belonging to different models on data with outliers. (3) We conduct the linkage clustering to generate only two clusters containing inlier cluster and outlier cluster to separate inliers and outliers for each selected model one by one, which is integrated into an alternate sampling and clustering framework.

The rest of this paper is organized as follows. In Section 2, we introduce the proposed method in detail. The experiments in multi-structure geometric model fitting, including multi-homography matrix estimation and multi-fundamental matrix estimation, are presented in Section 3. Finally, we draw our conclusions in Section 4.

## 2. Materials and Methods

The two-stage method conducted in this paper follows the classical framework for multi-model fitting, which firstly generates large amount hypotheses, then makes preference for the hypotheses, and finally segments the inliers belonging to different models. Both stages make use of the quantized residual and contain a linkage cluster process, the difference is that the objects used for clustering are not the same.

In the model selection stage, a large amount of hypotheses will be generated and the sum of several minimum residuals (hypothesis cost) will be calculated for every hypothesis to measure the quality of the hypotheses. Then quantized residual preference will be made for the hypotheses to propose linkage clustering, which is iteratively merging two hypotheses with minimum distance and updating with the one of smaller sum of residuals. Finally hypotheses retained with small hypothesis cost and considerable number of cluster members will be selected as the model selection results.

The inlier segmentation stage will be entered after the models are selected, quantized residual preferences are generated based on the initial inliers of the selected hypotheses for the points, which will be used to separate inliers and outliers for each selected models by linkage clustering. In addition, an alternate sampling and clustering framework is proposed to make sure optimum division of the inliers and outliers can be found.

In this section, we will first introduce how to calculate the quantized residual preference after generating several hypotheses for linkage clustering to select models, and then we will carefully explain how the quantized residual preference is used in linkage clustering under alternate sampling and clustering framework to separate the inliers and outliers for each selected model one by one.

### 2.1. Model Selection

**Figure 1:**
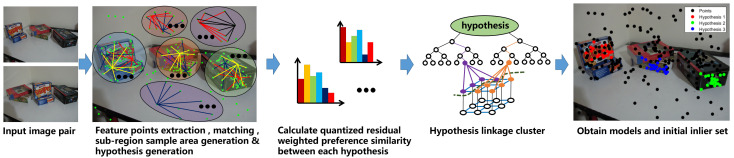
Model selection flow chart.

The model selection algorithm is proposed to obtain all the valid models in the scene. The flow chat of model selection is shown in Figure 1. Like most of the model fitting methods, a sampling process to generate a great number of hypotheses will be conducted firstly. In addition, in order to take advantage of the prior knowledge that inliers belonging to one model tend to be neighbours, the hypotheses generation follows the random sampling process, but within a region. All the data points are segmented into several subregions with the same size by Euclidean distance, and then hypotheses are generated by random sampling within each subregion.

Given the data point set $X=\{x_{1},x_{2},...,x_{N}\}$, the hypotheses set $H=\{\hbar^{1},\hbar^{2},\cdots ,\hbar^{j},\cdots ,\hbar^{M}\}$ after the hypothesis generation, and then the residual matrix $R=\{r^{1},r^{2},\cdots ,r^{j},\cdots ,r^{M}\}$, where $r^{j}={\left[r_{1,j},r_{2,j},\cdots ,r_{i,j},\cdots ,r_{N,j}\right]}^{T}$ refers to the residuals of hypothesis $\hbar^{j}$ to all the data points in *X*, *N* is the data number, and *M* is the number of hypotheses.

To calculate the hypothesis cost, we first need to sort the residuals of the hypothesis in ascending order. If ${\overset{´}{r}}^{j}=\left\{r_{\tau_{i},j}|r_{\tau_{1},j}\leq r_{\tau_{2},j}\leq \cdots \leq r_{\tau_{N},j},r_{\tau_{i},j}\in r^{j}\right\}$ is the ascending sorted residuals of hypothesis $\hbar^{j}$, then the hypothesis cost ${hc}_{j}$ of $\hbar^{j}$ is calculated by Equation (1).

(1) $${hc}_{j}=\sum_{i=1}^{k}r_{\tau_{i},j}$$

in which 1≤k≤N, and usually k=20.

Hypotheses with lower cost will be used in the quantized residual preference linkage clustering for selecting hypotheses with good quality. The quantized residual preference is actually the quantization on *R* by Equation (2).

(2) $$\begin{array}{c} {\overset{ˇ}{q}}_{i,j}=\left\lceil \frac{r_{i,j}-r_{min}^{j}}{r_{max}^{j}-r_{min}^{j}}*\theta \right\rceil \\ r_{max}^{j}=max\left\{r_{1,j},r_{2,j},\cdots ,r_{i,j},\cdots ,r_{N,j}\right\}\\ r_{min}^{j}=min\left\{r_{1,j},r_{2,j},\cdots ,r_{i,j},\cdots ,r_{N,j}\right\} \end{array}$$

where θ refers to the quantization level. When using the quantized residuals to represent the hypotheses or the data points, a valid quantization length λ is needed to decrease the complexity of the quantized residual preferences.

(3) $$q_{i,j}=\left\{\begin{array}{cc} {\overset{ˇ}{q}}_{i,j} & {\overset{ˇ}{q}}_{i,j}<=\lambda \\ 0 & {\overset{ˇ}{q}}_{i,j}>\lambda \end{array}\right.$$

In this way, we can obtain the quantized residual matrix $Q=\left[\begin{array}{ccc} q_{1,1} & \cdots & q_{1,M}\\ \vdots & \ddots & \vdots \\ q_{N,1} & \cdots & q_{N,M} \end{array}\right]$, where each column of *Q* is the quantized residual preference for the hypothesis, and each row of *Q* is the quantized residual preference for the data point. That is, the quantized residual preference for hypothesis $\hbar^{j}$ is the *j*th column of *Q*, i.e., $q^{j}={\left[q_{1,j},q_{2,j},\cdots ,q_{i,j},\cdots ,q_{N,j}\right]}^{T}$.

**Figure 2:**
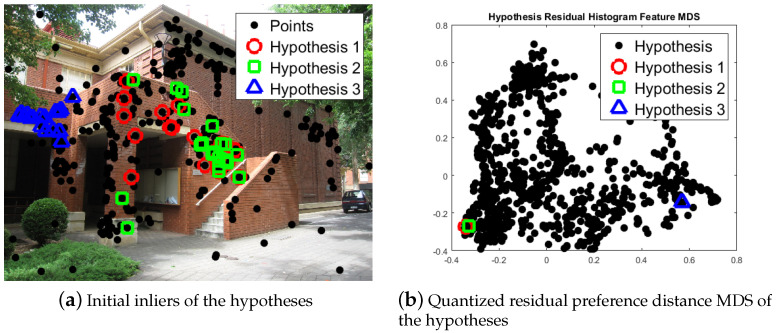
Hypotheses and their quantized residual preference distance (distance=1/WS+1) MDS. (**a**) The initial *k* inliers with the *k*th minimum residuals for the three hypotheses. (**b**) The quantized residual preference and the distance multidimensional scaling (MDS) points. Similar hypotheses (hypothesis 1 and hypothesis 2) are closer in the distance MDS, while a different hypothesis (hypothesis 3) is very far from hypothesis 1 and hypothesis 2.

Considering the impact of the inliers will be greater than the outliers for hypotheses, especially when comparing two quantized residual preferences. The quantized residual weighted preference similarity is defined by Equation (4), in which the more common elements between two quantized residual preferences, the more similar they are, and the smaller the common quantized value (except 0), the closer they are to a common model. A sample plot is presented in Figure 2 to show the effectiveness of weighted preference similarity for comparing two hypotheses.

(4) $$\begin{array}{c} W\left(q^{i},q^{j}\right)=\sum_{t=1}^{N}\psi \left(q_{i,t},q_{j,t}\right)\\ \psi \left(q_{i,t},q_{j,t}\right)=\left\{\begin{array}{cc} 1/q_{i,t} & \mathrm{if}\;q_{i,t}==q_{j,t},q_{i,t}\neq 0\\ 0 & \mathrm{else} \end{array}\right. \end{array}$$

The model selection process is actually a linkage clustering, which is aimed at clustering similar hypotheses and selecting hypotheses close to the model in represent of each cluster. When conducting linkage clustering, we iteratively merge the two hypotheses with the maximum similarity (minimum weighted preference distance) and update the similarity matrix and clusters, until the maximum similarity is less than a threshold. This threshold depends on the given valid quantization length. If the two hypotheses have only one common item at the end of the valid quantization length, then the two hypotheses are considered to be disrelated with high probability. Therefore, if the valid quantization length is taken as 20, then according to Equation (4), the threshold should be 0.05. For the similarity matrix updating, we preserve the similarity value of the hypothesis with the best quality (smaller hypothesis cost) and set the similarity values of the other hypotheses to 0 to avoid repetitive clustering. After the linkage clustering, models very different from each other are clustered into different classes, and hypotheses with the minimum hypothesis cost are left to represent each cluster. As there will also be some clusters consisting of bad hypotheses, we set a threshold(1% of the hypothesis number) for the size of cluster to remove these clusters by taking advantage of the random sample consensus, i.e., a good model will be more likely to be sampled repeatedly. The detailed model selection algorithm is presented in Algorithm 1.
**Algorithm 1**ModelSelection1: Calculate hypothesis cost for each hypothesis by Equation (1);2: Calculate quantized residual preference for hypotheses by Equations (2) and (3);3: Calculate the weighted preference similarity by Equation (4) between every two hypotheses, and obtained similarity matrix;4: Define each hypothesis as a cluster;5: Merge the two cluster with maximum weighted preference similarity into one cluster;6: Update the merged cluster with the quantized residual preference of smaller hypothesis, and replace the cluster similarity, while set the similarity of the other cluster to 0;7: Repeat from step 5, until the maximum weighted preference similarity is less ξ;8: remove the clusters whose size is less than 0.01∗hypothesis number.

### 2.2. Inlier Segmentation

The model selection process usually makes it possible for us to find all the models in the data set, except for the fact that the sampling is not sufficient. Meanwhile, through the model selection process, we just obtain the model inlier sets with a fixed size and the hypothesis clusters, and most of the time we need to obtain all the inliers of each model, so that we can perfectly separate the inliers and outliers for each model. As a result, inlier segmentation is proposed to obtain all the inliers of each model, under the circumstance that the parameters of each model are estimated. Similar to the model selection algorithm, it also includes a random sampling process and a hierarchical clustering operation. The difference is that the model selection algorithm randomly samples the sub-regions and clusters the hypotheses to obtain multiple models, while the inlier segmentation algorithm randomly samples the current inlier set and clusters each point to obtain all inliers belonging to each model.

**Figure 3:**
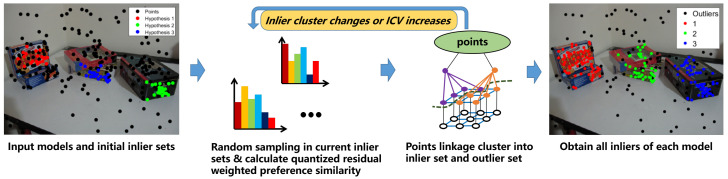
Inlier segmentation flow chart.

When we obtain the exact model parameters, a direct and easy way to separate the inliers is to set an inlier threshold to obtain the data points with residuals less than the threshold as the inliers. However, most of the time, this direct method works poorly, in which only some of the inliers can be separated. The exact true model parameters are very hard to find, and most of the time the parameters we find are only approximate, so the inliers within the threshold make it difficult to fully separate the inliers and outliers. In addition, when there is more than one model, one single threshold will not be enough to separate all the models’ inliers. Although some scale estimators claim to estimate the inlier scale, they have many limitations and require the noise distribution, which will usually fail in a real data set, and they work poorly when the model is complicated (such as homography matrix estimation and fundamental matrix estimation) and the data contain pseudo-outliers and noise. In contrast, a clustering method, taking advantage of the consensus representation, can separate the inliers and outliers without an inlier threshold or scale estimator. The use of the quantized residual preference for the hypotheses is very robust and efficient for linkage clustering in the model selection process to cluster similar hypotheses, and it can also be used to represent data points to separate the inliers from the outliers. The flow chat of inlier segmentation is shown in Figure 3.

For a better representation, we use the *k* points with minimum residuals of each selected model by Algorithm 1, to generate hypotheses and make quantized residual preference for the data points. We then conduct linkage clustering to generate only two clusters—inlier cluster and outlier cluster for each selected model one by one. When conducting inlier and outlier clustering, an iterative sampling and clustering framework is introduced to get a optimum result, which iteratively samples the hypotheses from the inlier cluster and extract the quantized residual preference for the points for inlier and outlier clustering, until the clustering result unchanged.

Given we get the selected models $\bar{H}=\left\{{\bar{h}}^{1},{\bar{h}}^{2},\cdots ,{\bar{h}}^{j},\cdots ,{\bar{h}}^{m}\right\}$ after model selection in Algorithm 1, and the residual matrix $\bar{R}=\left\{{\bar{r}}^{1},{\bar{r}}^{2},\cdots ,{\bar{r}}^{j},\cdots ,{\bar{r}}^{m}\right\}$ of all the selected models, where ${\bar{r}}^{j}={\left[{\bar{r}}_{1,j},{\bar{r}}_{2,j},\cdots ,{\bar{r}}_{i,j},\cdots ,{\bar{r}}_{N,j}\right]}^{T}$ refers to the residuals of model ${\bar{h}}^{j}$ to all the data points in *X*, *N* is the data number, and *m* is the number of selected models. Then for each selected model we collect its initial inlier set consisting of *k* points with minimum residuals. Since the proposed inlier segmentation is actually to separate the inliers from the outliers for each selected model one by one, the following will take model ${\bar{h}}^{j}$ as example to further explain our method.

When collecting the initial inlier set of model ${\bar{h}}^{j}$, firstly the residuals ${\bar{r}}^{j}$ of model ${\bar{h}}^{j}$ are sorted in ascending order ${\bar{r}}^{j}=\left\{{\bar{r}}_{\tau_{i}^{j},j}|{\bar{r}}_{{\bar{\tau }}_{1}^{j},j}\leq {\bar{r}}_{{\bar{\tau }}_{2}^{j},j}\leq \cdots \leq {\bar{r}}_{{\bar{\tau }}_{i}^{j},j}\leq \cdots \leq {\bar{r}}_{{\bar{\tau }}_{N}^{j},j},{\bar{r}}_{{\bar{\tau }}_{i}^{j},j}\in {\bar{r}}^{j}\right\}$. Then we collect ${\bar{\tau }}_{k}^{j}=\left[x_{{\bar{\tau }}_{1}^{j}},x_{{\bar{\tau }}_{2}^{j}},\cdots ,x_{{\bar{\tau }}_{k}^{j}}\right]$ as the initial inlier set for selected model ${\bar{h}}^{j}$.

**Figure 4:**
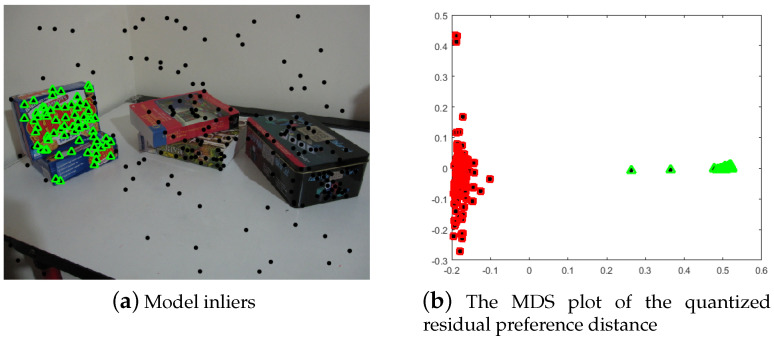
The inliers and their corresponding quantized residual preference MDS plot. (**a**) The inliers labeled with green triangles. (**b**) The corresponding quantized residual preference MDS plot and inliers (from (**a**)) marked with green triangles and outliers with red squares. The quantization level θ is 500, and the valid quantization length for preference λ is 20.

Then, several hypotheses will be generated through random sampling on initial inlier set ${\bar{\tau }}_{k}^{j}$, which will be soon used to make quantized residual preference for the data points the same way as the model selection process by Equations (2) and (3). As a consequence, more good hypotheses close to the model ${\bar{h}}^{j}$ will be used to produce the quantized residual preference for the data points, and it will make the quantized residual preferences for the inliers to have more smaller quantized values, and the quantized residual preferences for the outliers to have more bigger quantized values (or 0), which will make it possible to separate inliers from the outliers for model ${\bar{h}}^{j}$. Supposing we obtain the quantized residual preference matrix $\bar{Q}$, where each row of $\bar{Q}$ is the quantized residual preference for the data point. That is, the quantized residual preference for data point $x_{i}$ is the *i*th row of $\bar{Q}$, i.e., ${\bar{q}}_{i}=\left[{\bar{q}}_{i,1},{\bar{q}}_{i,2},\cdots ,{\bar{q}}_{i,j},\cdots ,{\bar{q}}_{i,\bar{m}}\right]$. When comparing two quantized residual preferences ${\bar{q}}_{i}$ and ${\bar{q}}_{j}$, the distance measurement defined by Equation (5) is used. Figure 4 presents the MDS plot of the quantized residual preference for the data points, from which we can see the inliers and outliers are well separated.

(5) $$\begin{array}{c} W\left(q_{i},q_{j}\right)=\left\{\begin{array}{cc} 1-\frac{\sum_{t=1}^{M}\varphi (q_{i,t},q_{j,t})}{max(\rho \left(q_{i}\right),\rho \left(q_{j}\right))} & \mathrm{if}max(\rho \left(q_{i}\right),\rho \left(q_{j}\right))\neq 0\\ 1 & \mathrm{else} \end{array}\right.\\ \varphi \left(q_{i,t},q_{j,t}\right)=\left\{\begin{array}{cc} 1 & \mathrm{if}q_{i,t}==q_{j,t},q_{i,t}\neq 0\\ 0 & \mathrm{else} \end{array}\right.\\ \rho \left(q_{i}\right)=\sum_{t=1}^{M}\varphi (q_{i,t},q_{i,t}) \end{array}$$

From Figure 4, we can see that the quantized residual preference for the points can separate the inliers from the outliers easily. In addition, we then undertake linkage clustering with a fixed cluster number of two to only cluster the inliers and outliers. To make the effect of the random hypothesis sampling in the inlier set stable and ensure that it can easily reach convergence, an iterative sampling and clustering framework is proposed to iteratively conduct the hypothesis sampling and linkage clustering. Furthermore, in order to avoid non-convergence and instability of the sampling, we use the inter-class variance (ICV) (Equation (6)) to measure the quality of the inlier cluster, i.e., good inlier separation presents bigger inter-class variance. Please note that $C_{I}$ refers inlier cluster and $C_{O}$ is outlier cluster, and ${\bar{r}}_{i}$ represents the residual of $x_{i}$ to the model calculated from the inlier set $C_{I}$ in Equation (6). The bigger the ICV value, the better the clustering result will be.

(6) $$\begin{array}{cc} ICV & =\frac{card\{C_{in}\}}{N}*\left(u_{in}-u\right)+\frac{card\{C_{out}\}}{N}*\left(u_{out}-u\right);\\ u & =\frac{1}{N}\sum_{i=1}^{N}r_{i};u_{in}=\frac{1}{card\{C_{in}\}}\sum_{x_{i}\in C_{in}}{\bar{r}}_{i};\\ u_{out} & =\frac{1}{card\{C_{out}\}}\sum_{x_{i}\in C_{out}}{\bar{r}}_{i}; \end{array}$$

Then the whole inlier segmentation process for model ${\bar{h}}^{j}$ under iterative sampling and clustering framework can be summarized. We first sample several hypotheses in the initial inlier set ${\bar{\tau }}_{k}^{j}$, and then extract the quantized residual preference $\bar{Q}$ and calculate the distance matrix for every two points. We undertake linkage clustering with a fixed cluster number of two to cluster the inliers $C_{in}$ and outliers $C_{out}$, and then calculate the inter-class variance by Equation (6). We then replace the initial inlier set ${\bar{\tau }}_{k}^{j}$ with inliers $C_{in}$, and again conduct hypothesis sampling on the inlier set in turn, this way we iteratively perform clustering then sampling, until the inlier set is unchanged or inter-class variance decreases. The detailed algorithm is presented in Algorithm 2.
**Algorithm 2**InlierSegmentation 1:Calculate residuals for selected model ${\bar{h}}^{j}$; 2:Collect initial inlier set; 3:Generate hypotheses on inlier set, and Calculate residuals; 4:Calculate quantized residual preference for data points and preference distance between every two data points; 5:Conduct linkage clustering to generate two clusters, take cluster more intersected with initial inlier set as inlier set; 6:Calculate the inter-class variance ICV; 7:**if** The inlier cluster unchanged or the inter-class variance ICV decreases **then** 8: go to step 12; 9:**else**10: Replace the initial inlier set with inlier set, and go back to step 3;11:**end if**12:Conduct step 1 to 11, until all the selected models are processed.

## 3. Experiment

In this section, we describe the experiments undertaken in multi-structure geometric model fitting, including multi-homography estimation and multi-fundamental matrix estimation, which are fundamental issues in image stitching [32] and visual localization [33]. Firstly, we describe the model selection results, and then the inlier segmentation results are presented. Comparisons on inlier segmentation with some of the state-of-the-art methods are made to present the characteristics of the proposed method.

**Figure 5:**
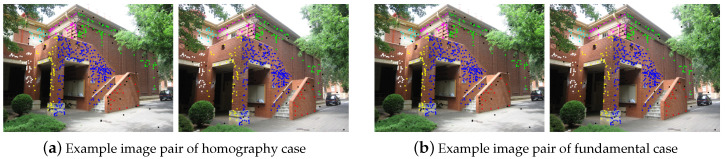
Example image pairs of the AdelaideRMF data set.

The AdelaideRMF [34] data set was used for the multi-homography and fundamental matrix estimation. Some image pairs of the data set is shown in Figure 5. The data set contains matching points in two uncalibrated images with gross outliers and the labels of the matching points are manual-annotated. In the first case (plane segmentation) the (static) scene contains several planes, each giving rise to a set of point correspondences described by a specific homography. The aim is to segment different planes by fitting homographies to subsets of corresponding points. In the second case (motion segmentation) the setup is similar, but the scene is not static, i.e., it contains several objects moving independently each giving rise to a set of point correspondences described by a specific fundamental matrix. The aim is to segment the different motions by fitting fundamental matrices to subsets of corresponding points. In addition to the fitting preference images, we also use the overall misclassification percentage (number of misclassified points divided by the number of all the points in the data set) [35] to present the fitting performance when dealing with the multi-homography and fundamental matrix estimation.

### 3.1. Multi-Homography Matrix Estimation

In this part, we describe the estimation of the multi-homography matrix by the use of the proposed method, and through the segmentation of the inliers belonging to the different homography models, we can segment the different planes. By using the same data as [14], we are able to compare the results directly to PEARL, SA-RCM, J-linkage, and T-linkage. The misclassification accuracies in Table 1 for the above four methods were obtained from [14].

**Figure 6:**
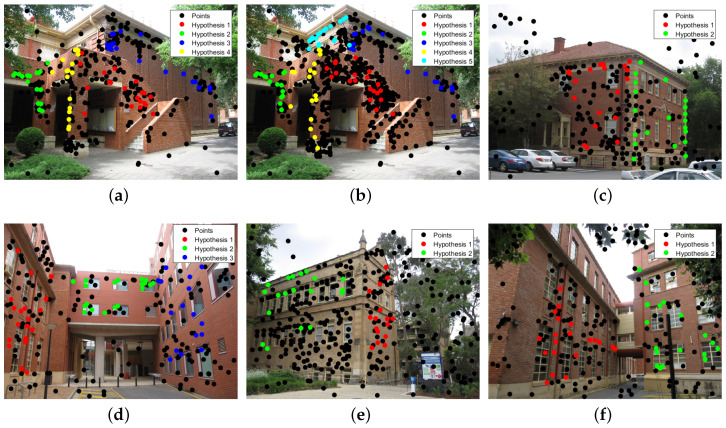
Model selection results for multi-homography matrix estimation. (**a**) johnsona. (**b**) johnsonb. (**c**) ladysymon. (**d**) neem. (**e**) oldclassicswing. (**f**) sene.

**Figure 7:**
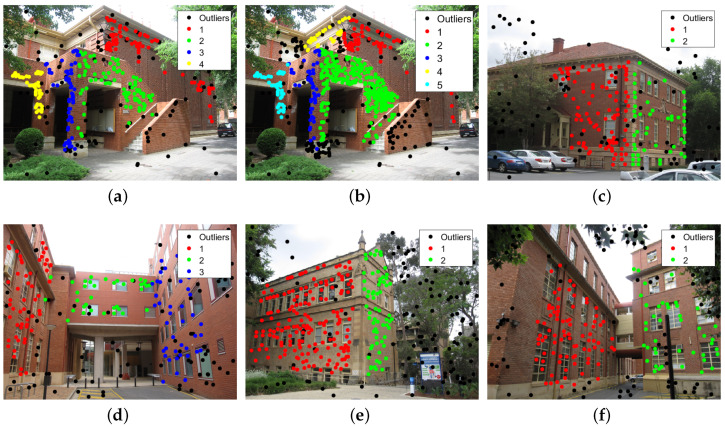
Results of the multi-homography matrix estimation of the proposed method. (**a**) johnsona. (**b**) johnsonb. (**c**) ladysymon. (**d**) neem. (**e**) oldclassicswing. (**f**) sene.

As can be seen in Figure 6, the proposed method can detect almost all the models, and the models are very close to the true models, except for the “johnsonb” data set. Five models are correctly detected, and the other two models with a few inliers are missed. From Figure 7, it can be seen that the proposed method can extract almost all the planes in the images, and the inlier points of the planes can be clearly classified. Except for the “johnsonb” image, five out of seven planes are extracted, while the other two planes with very fewer inliers are missed. Because the inliers are few in number and with many outliers and noise, it is very hard to obtain a good sampling hypothesis in these two areas. From the images, we can see that almost all the misclassified points are the points that are supposed to be inliers but are divided into outliers for all the models, and the misclassification is a result of the inliers not being fully extracted. Table 1 shows the misclassification results of the state-of-the-art methods and the proposed method, where it can be seen that the proposed method obtains the lowest misclassification result on the “johnsonb”, “ladysymon” and “sene” data sets, and the results on the other three data sets are also very close to the lowest misclassification result.

The above experiments on each data set were undertaken iteratively 20 times, and then the result with the minimum number of misclassified points was selected as the final result. The parameters for the multi-homography matrix estimation are quite easy to set. For the hypothesis generation, we set the minimum number of stable model inliers and the size of the sub-regions as k=20, the number of hypotheses randomly sampled from the sub-region as n=50, and the number of sub-regions $m\leq \left\lfloor \frac{N}{k}\right\rfloor$, where *N* is the number of points. In the model selection process (Algorithm 1), the quantization level θ was set as 500, the valid quantization length for building the histogram preference λ was set as 20, and the threshold δ to stop the linkage clustering was set to 0.05. In the inlier segmentation process (Algorithm 2), θ and λ were the same as in the model selection process (Algorithm 1), the cluster number Cn was set as 2, and the hypothesis number for sampling Hn was set as 100. Most of the time, the parameters in the model selection process (Algorithm 1) and the inlier segmentation process (Algorithm 2) do not need to be changed.

### 3.2. Multi-Fundamental Matrix Estimation

The proposed method was also used to estimate the multi-fundamental matrix in two-view images, and through the classification of the inliers belonging to the different fundamental models, different motions could be segmented. As in the multi-homography matrix estimation, the motion segmentation accuracy is compared to PEARL, SA-RCM, J-linkage, and T-linkage, and the misclassification accuracies in Table 2 for the above four methods were obtained from [14].

The experiments on each data set for the multi-fundamental matrix estimation were undertaken in the same way as the multi-homography matrix estimation experiments, i.e., the algorithm was iteratively conducted 20 times on one data set, and then the result with the minimum number of misclassified points was chosen as the final result.

**Figure 8:**
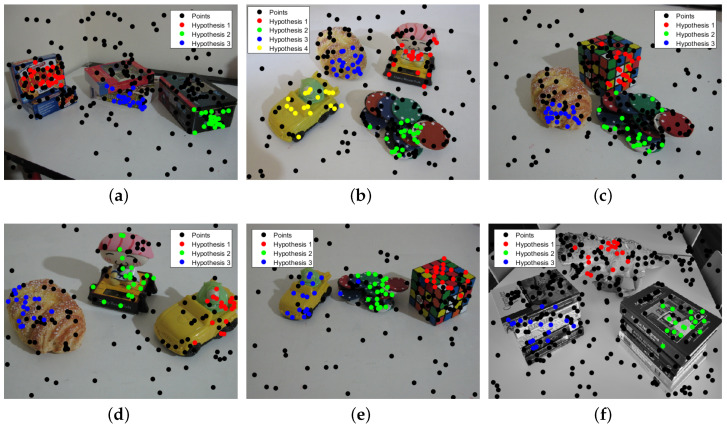
Model selection results for the multi-fundamental matrix estimation. (**a**) biscuitbookbox. (**b**) breadcartoychips. (**c**) breadcubechips. (**d**) breadtoycar. (**e**) carchipscube. (**f**) dinobooks.

**Figure 9:**
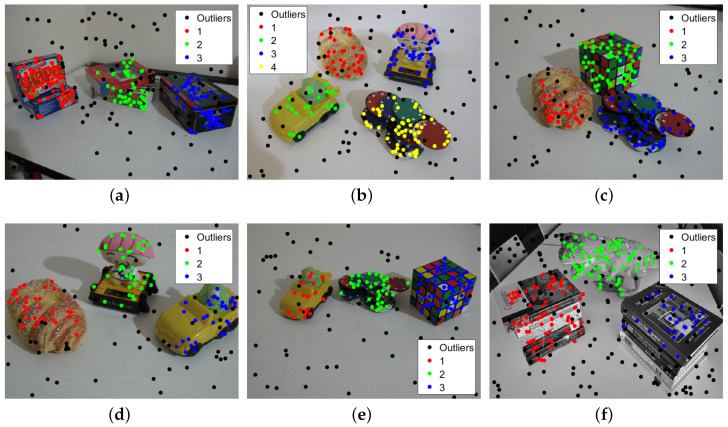
Inlier segmentation results for the multi-fundamental matrix estimation. (**a**) biscuitbookbox. (**b**) breadcartoychips. (**c**) breadcubechips. (**d**) breadtoycar. (**e**) carchipscube. (**f**) dinobooks.

From Figure 8, we can see that the proposed method performs quite well on the six images, and the motion models for the six images are correctly detected, except for the “carchipscube” data set, where the initial inlier set of the moving car contains two pseudo-outliers. Meanwhile, for the inlier segmentation in Figure 9, all the motions can be extracted and segmented, with few misclassified points, which can also be seen in the misclassification listed in Table 2. The proposed method performs better than the other four methods on five out of six images, especially on the “biscuitbookbox” and “carchipscube” data sets, where there are no misclassified points. The proposed method also reaches quite a close misclassification result when compared to the lowest misclassification result for the “breadcubechips” data set. From Figure 9, it can be seen that almost all the misclassified points are the inliers that are classified as outliers to all the models, and there are very few points that are classified as pseudo-outliers.

The parameters for the algorithm in these experiments needed a few changes when compared to the parameters used in the multi-homography estimation experiments. The number of randomly sampled hypotheses *n* needed to be increased for the larger size of MSS for the fundamental matrix estimation (at least eight points are needed to estimate a fundamental matrix), so we set n=80 for the multi-fundamental matrix estimation.

### 3.3. Computational Time Analysis

To further compare the performance of the algorithms, the computational time of ours in various scenarios is counted and compared with the T-Linkage method as the state-of-art. However, the computational time and specific values of parameters in different scenarios have not been given by the literature of the T-Linkage method, so we implemented the T-Linkage method according to [14], achieved similar accuracy to [14] by adjusting the parameters and then counted the calculation time. When comparing with the T-Linkage method in computational time, we found that the it is closely related to the number of points and models, the ratio of outliers, and the value of the thresholds. The detail results can be seen in Table 3 and Table 4. Both methods run in MATLAB and the environment of hardware is i7-9017, Core8 and 16G RAM.

From the above two tables, it can be found that in most cases, T-Linkage method takes less time than ours. This is mainly because that our method performs random sampling and linkage clustering in both two stages, and uses an alternate sampling and clustering strategy in the inlier segmentation stage to eliminate the interference from outliers, which will increase the computational time. However, our algorithm basically does not require parameter adjustment in various scenarios, which can save a lot of time for parameter adjustment and improve the applicability of the algorithm.

### 3.4. Computational Complexity Analysis

The computational complexity of the multi-model fitting algorithms mainly exists in the need for a large number of sampling and the calculation of the similarity between every two points for clustering. Since the value of each parameter is not given in the literature of the T-Linkage method, it is difficult to quantitatively evaluate the computational complexity, but we can give a qualitative analysis.

When calculating the similarity value, a continuous exponential function is used by T-Linkage method to describe the distance from the point to the model, mapping the residual between 0 and 1. However, the quantized residual is used by our algorithm to describe the distance between the point and the model. It only needs to find the maximum and minimum residuals and divide the remaining residuals according to the quantization level, and then take the valid quantization length for subsequent processing. Therefore, the computational complexity here is significantly lower than the T-Linkage method.

However, in terms of sampling, our two-stage method is significantly more complex than T-Linkage method. In the model selection stage, we randomly sample in the sub-region of the scene, generate a large number of hypotheses and cluster the hypothesis models to get all the valid models. Next, in the inlier segmentation stage, we randomly sample the current inlier set of each model, generate a large number of hypotheses, and cluster the points to obtain all the inliers of each model. Unlike our method, after random sampling and obtaining a large number of hypotheses, the T-Linkage method only clusters the points which needs to adjust the threshold of inliers for each application case.

In general, our two-stage method has less computational complexity when calculating similarity and greater computational complexity in the number of random samples. Since random sampling is required in both stages, the computational complexity of our method is greater than T-Linkage method in total.

## 4. Conclusions

In this paper, we have proposed a robust two-stage multi-model fitting method, which is composed of model selection and inlier segmentation. During the model selection, the quantized residual preference is extracted for the hypothesis linkage clustering to obtain the main structure models in the data. The inlier segmentation process is then performed as an iterative sampling and clustering process using the quantized residual preference of the points. The experimental results show that the model selection method can successfully detect models that are very close to the true models. Furthermore, the inlier segmentation method can separate the inliers from the outliers for the different models, and the proposed method outperforms the state-of-the-art methods in multi-structure geometric model fitting.

## Figures and Tables

**Table 1:** Misclassification (%) for the multi-homography matrix estimation.

Methods	PEARL	J-Linkage	T-Linkage	SA-RCM	Proposed
johnsona	4.02	5.07	4.02	5.90	5.09
johnsonb	18.18	18.33	18.33	17.95	14.18
ladysymon	5.49	9.25	5.06	7.17	1.69
neem	5.39	3.73	3.73	5.81	4.15
oldclassicswing	1.58	0.27	0.26	2.11	0.79
sene	0.80	0.84	0.40	0.80	0.40

**Table 2:** Misclassification (%) for the mullti-fundamental matrix estimation.

Methods	PEARL	J-Linkage	T-Linkage	SA-RCM	Proposed
biscuitbookbox	4.25	1.55	1.54	7.04	0
breadcartoychips	5.91	11.26	3.37	4.81	0.84
breadcubechips	4.78	3.04	0.86	7.85	0.87
breadtoycar	6.63	5.49	4.21	3.82	1.20
carchipscube	11.82	4.27	1.81	11.75	0
dinobooks	14.72	17.11	9.44	8.03	7.22

**Table 3:** Computational time of T-Linkage and our method in each homography scene.

Scene	Points	Models	Outlier Rate (%)	Methods	Number of Samples	Computational Times (ms)
johnsona	373	4	20.91	T-Linkage	2000	346
				Ours	950	473
johnsonb	649	7	12.02	T-Linkage	4000	977
				Ours	1650	953
ladysymon	237	2	32.49	T-Linkage	2000	249
				Ours	600	284
neem	241	3	36.51	T-Linkage	2000	281
				Ours	600	305
oldclassicswing	379	2	32.45	T-Linkage	2000	322
				Ours	950	387
sene	250	2	47.2	T-Linkage	2000	293
				Ours	650	314

**Table 4:** Computational time of T-Linkage and our method in each fundamental scene.

Scene	Points	Models	Outlier Rate (%)	Methods	Number of Samples	Computational Times (ms)
biscuitbookbox	259	3	37.45	T-Linkage	2000	305
				Ours	1040	463
breadcartoychip	237	4	34.6	T-Linkage	2000	287
				Ours	960	431
breadcubechips	230	3	35.22	T-Linkage	2000	284
				Ours	960	405
breadtoycar	166	3	33.73	T-Linkage	2000	213
				Ours	720	316
carchipscube	165	3	36.36	T-Linkage	2000	204
				Ours	720	313
dinobooks	360	3	43.06	T-Linkage	2000	473
				Ours	1440	549
